# The individual environment, not the family is the most important influence on preferences for common non-alcoholic beverages in adolescence

**DOI:** 10.1038/s41598-017-17020-x

**Published:** 2017-12-04

**Authors:** Andrea D. Smith, Alison Fildes, Suzanna Forwood, Lucy Cooke, Clare Llewellyn

**Affiliations:** 1 Department of Behavioural Science and Health, University College London, London, United Kingdom; 2 School of Psychology, University of Leeds, Leeds, United Kingdom; 3 Department of Psychology, Anglia Ruskin University, Cambridge, United Kingdom; 4 Great Ormond Street Hospital, Children NHS Foundation Trust, London, United Kingdom

## Abstract

Beverage preferences are an important driver of consumption, and strong liking for beverages high in energy (e.g. sugar-sweetened beverages [SSBs]) and dislike for beverages low in energy (e.g. non-nutritive sweetened beverages [NNSBs]) are potentially modifiable risk factors contributing to variation in intake. Twin studies have established that both genes and environment play important roles in shaping food preferences; but the aetiology of variation in non-alcoholic beverage preferences is unknown. 2865 adolescent twins (18–19-years old) from the Twins Early Development Study were used to quantify genetic and environmental influence on variation in liking for seven non-alcoholic beverages: SSBs; NNSBs; fruit cordials, orange juice, milk, coffee, and tea. Maximum Likelihood Structural Equation Modelling established that beverage preferences have a moderate to low genetic basis; from 18% (95% CI: 10%, 25%) for orange juice to 42% (36%, 43%) for fruit cordials. Aspects of the environment that are *not* shared by twin pairs explained all remaining variance in drink preferences. The sizeable unique environmental influence on beverage preferences highlights the potential for environmental modification. Policies and guidelines to change preferences for unhealthy beverages may therefore be best directed at the wider environment.

## Introduction

Beverages are increasingly becoming substantial contributors to individual energy intake as the availability and diversity of sugar-sweetened beverages (SSBs), fruit juices and other calorie-containing beverages continues to grow^1^. There is considerable concern about the increased consumption of SSBs, mainly because calorically-sweetened liquids have a weak satiety effect, which may result in poor energy compensation, and the development of overweight^2^. Negative health consequences are not limited to an increased risk of obesity, but also include the comorbidities associated with obesity – such as type 2 diabetes, cardiovascular disease and raised risk for various cancers – and dental caries. Global beverage consumption patterns have evolved in the past decades with a marked increase in the consumption of SSBs, although it appears that the market share for non-nutritive sweetened beverages (NNSBs) is gradually growing in importance. Purchases of lower sugar, zero calories soft drinks have for the first time overtaken regular soft drinks as the most popular soft drink type in the UK^3^. Nevertheless, the overarching shift towards greater consumption of SSBs, fruit juices and energy drinks among adolescents, in particular in low- to middle-income countries, highlights this as a key population at risk of the detrimental health risks associated with frequent consumption of energy-dense beverages^4–6^.

Preferences are important drivers of actual intake insofar as we tend to eat and drink what we like most. Stronger liking for beverages high in energy and decreased liking for beverages low in energy may therefore be important contributors to variation in intake. Preference for energy-dense beverages during adolescence may be especially detrimental to energy balance as consumption of these beverages peaks during this developmental period^7^.

Understanding what shapes preferences for different types of beverages is a crucial first step towards the development of interventions to modify them^8^. Twin studies are a powerful method for understanding the aetiology of behaviors and cognitions, because they establish the relative importance of genetic and environmental influences on variation in any given trait. Previous twin studies have established that individual differences in preferences for a range of foods have a moderate genetic basis in children (54% for vegetables, 27% for dairy and protein foods, and 20% for dessert foods)^9,10^, adolescents (54% for vegetables, 43% for snack foods, 54% for ‘healthy’ foods, and 39% for meat)^11,12^ and adults (30–38% for high fat, salt and sugar foods, 40% for ‘healthful’ foods, 58% for ‘distinctive’ tastes, and 36% for fruit and vegetables)^13,14^; yet the aetiologies of preferences for a variety of non-alcoholic beverages are yet to be established. Two previous twin studies examined genetic and environmental influences on preferences for coffee^15,16^, finding moderate genetic influence (42% in Luciano *et al*., 2005 and 62% in Vink *et al*., 2009). However, both studies defined coffee preference as a relative preference to tea, indexed as the ratio of number of cups of coffee to tea, consumed per day, rather than absolute liking of coffee per se.

Food and beverage preferences may differ substantially with respect to their aetiology. There is a need for studies to investigate the relative contribution of genetic and environmental factors to variation in preferences for a range of different types of beverages to inform where best to direct public health initiatives aimed at decreasing consumption of energy-dense beverages, especially among adolescents. The objective of this study was therefore to establish the relative importance of genetic and environmental influences on preferences for a range of non-alcoholic beverages among older adolescents.

## Subjects and Methods

### Sample

Participants were drawn from the Twins Early Development Study (TEDS), a population-representative cohort of 16,810 families with twins born in England and Wales in 1994–96^17^. The TEDS sample is largely representative of the UK population^17^. For the current study, twins were from a subsample of pairs born between September 1995 and August 1996. Invitations to complete an online beverage preference questionnaire were sent by letter and e-mail to the entire sub-sample (3166 pairs; n = 6332 individuals). Participation was rewarded with a £10 voucher to complete the survey and entry into a prize draw to win a pair of iPad Minis. Entry into the prize draw was conditional on completion of the questionnaire by the co-twin, to encourage responses from complete twin pairs. Participants provided informed consent. Procedures followed were in accordance and approved by the King’s College London Institute of Psychiatry ethics committee.

3155 of 6332 invited (49.8%) individuals agreed to participate in the current study. Of those, 290 were excluded due to medical- or perinatal problems, or due to unknown sex or zygosity. The final sample consisted of 2865 individuals, representing 1010 monozygotic twin individuals, 908 dizygotic same-sex (DZss) twin individuals, and 947 DZ opposite-sex (DZos) individuals. Of the excluded participants, 52 (17.9%) were MZs, 156 (58.6%) were DZs and 82 (21.8%) were of unknown zygosity. Excluded individuals did not differ significantly by zygosity (F (1, 3496) = 7.01, p = 0.08) or for factors that may be linked with beverage preferences such as health status (χ² = 5.918, p = 0.15) or BMI (t = 0.45, p = 0.65).

## Measures

### Sociodemographic measures and zygosity

Basic demographic information had been collected at first contact (age 18 months), including data on date of birth, sex, birth- or medical complications and socioeconomic status. Participants reported current height and weight, which was used to compute body mass index (BMI), calculated by dividing weight by height squared (kg/m^2^). Zygosity of same-sex twin pairs had previously been assigned using a parent-rated similarity questionnaire, validated by DNA analysis and shown to be >95% accurate^18^. Pairs for whom zygosity was uncertain had their zygosity determined by DNA genotyping, if DNA was available.

### Beverage preferences

As part of a wider study focused on studying food preference patterns in adolescence, seven beverage types were included in a comprehensive online self-report food preference questionnaire. Participants were instructed to rate their liking for sugar-sweetened beverages (SSBs) (‘non-diet fizzy beverages (e.g. Coca Cola, Pepsi)’), non-nutritive sweetened beverages (NNSBs) (‘diet fizzy beverages (e.g. Diet Coke, Pepsi Max)’), fruit cordial (‘Ribena or other fruit squash (e.g. orange squash)’), ‘orange juice’, ‘milk’, ‘tea (unsweetened)’, and ‘coffee (unsweetened)’ on a 5-point Likert scale, anchored by ‘not at all’ to ‘a lot’. For the ‘tea’ and ‘coffee’ items, separate questions enquired about preferred add-ins (e.g. addition of milk or any type of sweetness), but these preferences were not included in the present study. Higher scores indicated greater liking of that type of beverage. Paired-samples t-tests were used to compare mean group preferences for all seven beverage types. For any beverage types that had never been tasted or were unfamiliar, participants were instructed to select a ‘Not applicable’ option.

Test-retest reliability of the beverage preference questionnaire was assessed over a two-week period using a sample of the twins’ siblings. Of the 205 siblings invited to complete the online test-retest of the beverage questionnaire, 94 siblings provided responses at both rounds of data collection. Mean beverage preference test-retest scores indicated that responses were reliable over a 2-week period, with test-retest coefficients as follows: 0.69 for tea, 0.72 for orange juice, 0.77 for coffee, 0.85 for milk, 0.86 for SSBs, 0.89 for fruit cordial, and 0.93 for NNSBs.

### Statistical analysis

Twin analyses were used to estimate genetic and environmental influence on variation in preference for each of the seven beverage types.

The basis of the twin method is to compare the degree of resemblance between identical (monozygotic, MZ) pairs who share 100% of their genes, with that between non-identical (dizygotic, DZ) pairs who share approximately 50% of their segregating genes. The more similar MZ pairs are for a trait than DZ pairs, the stronger the genetic contribution to that trait. This inference is based on the assumption that MZ twins are twice as similar genetically compared to DZs, but both types of twins share their environments to a very similar extent^19^. The statistic derived to estimate the genetic contribution is called ‘heritability’ (A; additive genetic influences), and can be thought of as an index of the genetic effect size; heritability quantifies the proportion of trait variation attributable to genetic variation. As well as estimating genetic influence, the twin method partitions sources of environmental influence into: (i) aspects that are completely shared by both twins and contribute to their similarity, such as the home environment, SES and schooling experiences (‘shared environmental influences’); and (ii) and aspects that are unique to each twin in a pair and contribute to differences between pairs, such as having different friends or bouts of illness experienced by only one of the twins (‘unique environmental influences’). Variation is therefore attributed to three components of variance: additive genetic influences (A), shared environmental influences (C) and non-shared environmental influences (E). The unique environmental component of variance also includes random measurement error.

Intra-class correlations (ICCs) were calculated for beverage preference scores for MZ and DZ pairs to provide an indication of the pattern of similarity for the two types of twins. Maximum Likelihood Structural Equation Modelling (MLSEM) was used to derive precise estimates of A, C and E (with 95% confidence intervals and goodness-of-fit statistics) based on the expected structure of the variance-covariance matrices for MZs and DZs, based on the assumptions of genetic relatedness and equal environments (e.g. MZ covariation reflects the fact that they share all of their genes and all of their shared environments, rMZ = 1 A + 1 C; on the other hand, DZ covariation reflects the fact that they share half of their genes, but all of their shared environments, rDZ = 0.5 A + 1 C). Non-additive genetic effects, denoted by ‘D’, can also be investigated in a separate model that includes D instead of C (A, D and E), but D and C cannot be estimated in the same model with twins only. MZ ICCs that are greater than twice the DZ ICCs (ICC_MZ_ > 2 ICC_DZ_) indicate non-additive genetic effects contributing to variation. Because beverage preference scores were skewed, an alternative approach to deal with such variables is to summarize preference scales and to dichotomize them by the median value of the scale. Instead of ICC’s, tetrachoric correlation coefficients (TTC’s) are calculated to estimate phenotypic concordance rates for both types of twins.

Preference ratings for each of the beverage types were regressed on age and sex prior to modelling because twins share their age exactly (and sex for same-sex twins), and these factors can therefore inflate the shared environmental effect^20^. Prior to estimating A, C and E, a saturated model was fitted which applies no constraints to the data, and simply estimates means, covariances and variances for MZs and DZs. The model specifying A, C and E (ACE model) was then compared to the saturated model for goodness-of-fit, using the Likelihood Ratio Test (LRT) and the Akaike Information Criterion (AIC). According to the LRT a significant change in fit from the saturated model to a specified model indicates worsening of fit; a non-significant change indicates that the ACE model fits the data well (i.e. no significant change in fit). A lower AIC value indicates a better fitting model, with a difference of >2 indicating a superior model (AIC was also used for comparing non-nested models; e.g. to compare an ACE to an ADE model). Submodels that dropped A or C or A and C were then tested against the full ACE model to find a more parsimonious model. Just as for continuous traits, a liability threshold model was used to estimate A, C, and E for categorical (i.e. dichotomized) data. Sex-limitation models were also tested for each beverage type, to establish if there were sex-specific effects. These models test whether the magnitude of A, C and E differ for males and females (*quantitative sex-differences*), and whether the genetic and environmental influences are the same or different for males and females (*qualitative sex differences*)^21^. MLSEM was performed in R^22^, using the structural equation modelling software OpenMx, version 2.2.6^23^. The datasets generated during and analysed during the current study are available from the corresponding author on reasonable request.

## Results

Characteristics of the sample are shown in Table 1. Mean age of the sample was 19.1 years (SD = 0.3), and the sample was reasonably lean (mean BMI = 22.3 kg/m^2^). 40.2% of participants were male, and the MZ/DZ ratio (MZ pairs = 35.3%) reflected that of the general European twin population (roughly 1:2)^24^.

**Table 1:** Demographic characteristics of the study sample (n = 2865).

Characteristic	Sample	
	[*n* (%)]	
Sex		
M	1152	(40.2)
F	1713	(59.8)
Zygosity		
MZ^1^	1010	(35.3)
DZ^1^	1855	(64.7)
Age (SD)	19.1	(0.3)
BMI (SD)	22.3	(4.2)

^1^Abbreviations: MZ = Monozygotic; DZ = Dizygotic.

Table 2 summarizes mean preference scores for each beverage type. Orange juice was the most popular beverage, with the highest mean preference score, and NNSBs were the least liked indicated by the lowest mean score. There were significant differences in mean preference scores across all beverage pairings (p < 0.01) apart from between liking for fruit cordial ([4.23 (SD = 1.02)] and milk [4.22 (SD = 0.95)], t(2702) = 0.166, p = 0.87).

**Table 2:** Beverage preference scores and intraclass correlations (ICC) by zygosity.

**Beverage item**	**n** ^1^ (**%**)^2^	**Mean preference score** ^3^ (SD)	**MZ** ^4^ **ICC** ^4^ (**95% CI**)		**DZ** ^4^ **ICC** ^4^ (**95% CI**)	
**SSBs** ^4^	2841 (99.2)	3.73 (1.37)	0.383	(0.301, 0.458)	0.155	(0.085, 0.223)
**NNSBs** ^4^	2827 (98.7)	3.64 (1.34)	0.393	(0.221, 0.384)	0.222	(0.155, 0.288)
**Orange juice**	2849 (99.4)	4.43 (0.97)	0.261	(0.174, 0.345)	0.000	(0.000, 0.051)
**Fruit cordial**	2847 (99.3)	4.23 (1.02)	0.422	(0.344, 0.494)	0.212	(0.142, 0.280)
**Milk**	2707 (94.5)	4.22 (0.95)	0.377	(0.289, 0.457)	0.091	(0.013, 0.168)
**Tea**	2415 (84.3)	4.31 (1.08)	0.532	(0.450, 0.604)	0.000	(0.000, 0.066)
**Coffee**	1905 (66.5)	3.85 (1.29)	0.341	(0.214, 0.453)	0.069	(0.000, 0.172)

^1^Number of observations included in mean beverage liking score (excl. observations from individuals that never consume the specific beverage).

^2^Percentage of the full sample that reported occasional consumption of the beverage.

^3^Preference scores were rated on a 5-point Likert scale, with a higher score indicating a higher preference for the beverage item.

^4^Abbreviations: ICCs: Intraclass Correlations; MZ: Monozygotic; DZ: Dizygotic; NNSBs: Non-nutritive sweetened beverages, SSB: Sugar-sweetened beverages.

The MZ and DZ intraclass correlations for the seven beverage types are also shown in Table 2. For each beverage type within-pair correlations (ICCs) for MZs were moderate and higher than for DZs, indicating a genetic contribution to preferences for each type of beverage. For liking of SSBs, orange juice, milk, tea, and coffee the MZ ICCs were more than twice the magnitudes of the DZ ICCs.

**Figure 1:**
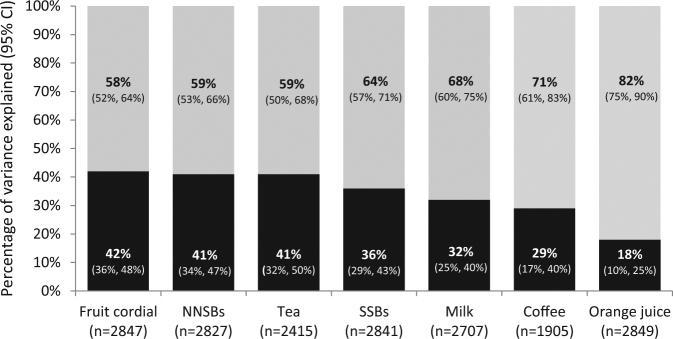
Genetic and environmental influences for the preference of seven non-alcoholic beverages. ^1^Estimates of the percentage of variance in beverage preferences explained by genetic (black portions of bars) and environmental (gray portions of bars) factors in 2865 participants from the Twins Early Development Study, aged 18–19 years.

MLSEM was used to derive A, C and E for each beverage type. Heritability estimates (A) across all beverage types were moderate to low for each of: fruit cordial (0.42; 95% CI: 0.36, 0.43), NNSBs (0.41; 95% CI: 0.34, 0.47), tea (0.41; 95% CI: 0.32, 0.50), SSBs (0.36; 95% CI: 0.29, 0.51), milk (0.32; 95% CI: 0.25, 0.40), coffee (0.29; 95% CI: 0.17, 0.40), and orange juice (0.18; 95% CI: 0.10, 0.25). The 95% confidence intervals demonstrated that genetic influences were significantly higher for liking of SSBs, NNSBs, tea and fruit cordial, than for liking of orange juice. No significant influence of the shared environment was observed for liking of any of the beverages, with the remaining variance being explained by environmental effects unique to each individual twin. AE models (that dropped the C component of variance) were therefore preferred for each beverage type. The relative contribution of genetic and environmental influences on individual beverage preferences are shown in Fig. 1, and the ACE model results with goodness-of-fit statistics are shown in Table 3.

**Table 3:** Model fit and parameter estimates for the saturated, ACE model and submodels of beverage preferences.

Beverage type	Additive genetic effect (A)	Shared environment effect (C)	Nonshared environment effect (E)	-2LL^3^	Df^3^	AIC^3^	Δ -2LL	p-value
**SSBs** ^**3**^								
Sat				9609.825	2832	3945.825		
ACE^1^	0.36 (0.26, 0.43)	0.00 (0.00, 0.09)	0.64 (0.57, 0.71)	9614.843	2835	3944.843	5.018	0.170
**AE** ^**2**^	**0.36 (0.29, 0.43)**	**—**	**0.64 (0.57, 0.71)**	**9614.843**	**2836**	**3942.843**	**0**	**1**
CE^2^	—	0.24 (0.19, 0.29)	0.76 (0.71, 0.81)	9631.547	2836	3959.547	16.704	< 0.001
E^2^	—	—	1.00 (1.00, 1.00)	9703.572	2837	4029.572	72.025	< 0.001
**NNSBs** ^**3**^								
Sat				9545.841	2818	3909.841		
ACE^1^	0.35 (0.15, 0.47)	0.05 (0.00, 0.20)	0.60 (0.55, 0.68)	9546.322	2821	3904.322	0.481	0.923
**AE** ^**2**^	**0.41 (0.34, 0.47)**	**—**	**0.59 (0.53, 0.66)**	**9546.719**	**2822**	**3902.719**	**0.397**	**0.529**
CE^2^	—	0.28 (0.23, 0.33)	0.72 (0.67, 0.77)	9557.576	2822	3913.576	11.254	< 0.001
E^2^	—	—	1.00 (1.00, 1.00)	9660.699	2823	4014.699	114.377	< 0.001
**Orange juice**								
Sat				7844.431	2840	2164.431		
ACE^1^	0.18 (0.09, 0.25)	0.00 (0.00, 0.04)	0.82 (0.75, 0.90)	7862.541	2843	2176.541	18.11	< 0.001
**AE** ^**2**^	**0.18 (0.10, 0.25)**	**—**	**0.82 (0.75, 0.90)**	**7862.541**	**2844**	**2174.541**	**0**	**1**
CE^2^	—	0.08 (0.03, 0.14)	0.92 (0.86, 0.97)	7873.266	2844	2185.266	10.725	0.001
E^2^	—	—	1.00 (1.00, 1.00)	7881.523	2845	2191.523	18.982	< 0.001
**Fruit cordial**								
Sat				8031.215	2838	2355.215		
ACE^1^	0.42 (0.23, 0.48)	0.00 (0.00, 0.15)	0.58 (0.52, 0.90)	8034.727	2841	2352.727	3.512	0.319
**AE** ^**2**^	**0.42 (0.36, 0.48)**	**—**	**0.58 (0.52, 0.64)**	**8034.727**	**2842**	**2350.727**	**0**	**0.998**
CE^2^	—	0.29 (0.24, 0.34)	0.71 (0.66, 0.76)	8051.672	2842	2367.672	16.945	< 0.001
E^2^	—	—	1.00 (1.00, 1.00)	8157.514	2843	2471.514	122.787	< 0.001
**Milk**								
Sat				7269.105	2698	1873.105		
ACE^1^	0.32 (0.25, 0.40)	0.00 (0.00, 0.06)	0.68 (0.60, 0.75)	7281.350	2701	1879.350	12.245	0.007
**AE** ^**2**^	**0.32 (0.25, 0.40)**	**—**	**0.68 (0.60, 0.75)**	**7281.350**	**2702**	**1877.350**	**0**	**1**
CE^2^	—	0.20 (0.14, 0.26)	0.80 (0.74, 0.86)	7298.526	2702	1894.526	17.176	< 0.001
E^2^	—	—	1.00 (1.00, 1.00)	7340.874	2703	1934.874	59.524	< 0.001
**Tea**								
Sat				7098.183	2406	2286.183		
ACE^1^	0.41 (0.32, 0.50)	0.00 (0.00, 0.03)	0.59 (0.50, 0.68)	7134.002	2409	2316.002	35.82	< 0.001
**AE** ^**2**^	**0.41 (0.32, 0.50)**	**—**	**0.59 (0.50, 0.68)**	**7134.002**	**2410**	**2314.002**	**0.00**	**1**
CE^2^	—	0.19 (0.12, 0.26)	0.81 (0.74, 0.88)	7171.829	2410	2351.829	37.83	< 0.001
E^2^	—	—	1.00 (1.00, 1.00)	7201.412	2411	2379.412	67.41	< 0.001
**Coffee**								
Sat				6351.94	1896	2559.94		
ACE^1^	0.29 (0.12, 0.39)	0.00 (0.00, 0.11)	0.71 (0.61, 0.83)	6358.921	1899	2560.921	6.9809	0.07
**AE** ^**2**^	**0.29 (0.17, 0.40)**	**—**	**0.71 (0.61, 0.83)**	**6358.921**	**1900**	**2558.921**	**0**	**1**
CE^2^	—	0.17 (0.09, 0.25)	0.83 (0.75, 0.91)	6366.625	1900	2566.625	7.7045	< 0.001
E^2^	—	—	1.00 (1.00, 1.00)	6382.128	1901	2580.128	23.208	< 0.001

Maximum Likelihood Structural Equation Modelling (MLSEM) was used to derive estimates of A, C and E, as well as provide two goodness-of-fit statistics; -2LL and the AIC respectively. The selection of the most parsimonious model was indicated by the p-value and the lowest absolute value of the AIC.

^1^The full ACE model was nested within the saturated model.

^2^Sub-models were nested within the full ACE model.

^3^Abbreviations; *- 2LL*: *-2 times log-likelihood of data*, *df*: *degrees of freedom*, *AIC*: *Akaike Information Criterion* (*AIC*), *NNSBs*: *Non-nutritive sweetened beverages*, *SSB*: *Sugar-sweetened beverages*.

Because the MZ ICCs were greater than twice the DZ ICCs for SSBs, orange juice, milk, tea, and coffee preference scores, suggestive of some non-additive genetic effects, ADE models were also examined and compared to ACE models (Supplemental Table 1). For liking of orange juice, milk and tea, the ADE model provided a better fit than the ACE model (*Δ*AIC = 7.8, 4.07 and 24.19 respectively), but no significant non-additive genetic effects (D) were found. AE models therefore provided the most parsimonious solutions across all beverage types. Similarly, because scale scores were skewed for orange juice, milk, tea and coffee, threshold models (treating beverage preference scores as binary traits) were considered as well as the models of continuous data. However, the genetic and environmental aetiology estimates from the threshold models did not differ significantly from the results shown in Table 3. Due to the loss in statistical power to estimate genetic variance components accurately when a trait is modelled as a binary rather than a continuous variable, results from the liability threshold models are shown in the supplemental materials (Supplemental Tables 2 and 3).

Similarly, results from the sex-limitation models found no strong evidence of significant sex differences. Parameters estimates and goodness-of-fit statistics for the full qualitative and quantitative sex-limitation models are shown in Supplemental Tables 4–11.

## Discussion

### Summary of findings

This is the first paper to establish the relative importance of genetic and environmental influences on liking for a range of non-alcoholic beverages, in a large population-based sample of older adolescent twins. Liking for SSBs, NNSBs, fruit cordial, orange juice, milk, coffee, and tea are entirely shaped by genetic and *unique* environmental influences in this age group. There was no detectable influence of the shared environment, which includes aspects of the environment completely shared by twin pairs; such as growing up in the same home, having the same parents, or shared schooling experiences. This suggests that shared environmental factors are unimportant in shaping preferences for non-alcoholic beverages for older adolescents, a developmental period characterised by a marked increase in autonomy in relation to dietary behaviour.

Similar to previous studies on the aetiology of food preferences, variation for the liking of seven beverage types was found to have a small to moderate genetic basis. Two previous studies have investigated the genetic and environmental contributions to *relative* liking for coffee (over tea), showing that approximately 42% to 62% of variation in coffee preference was attributable to genetic differences between adults^15,16^. These estimates are at the higher end of those observed in the current study (ranging from 18% for orange juice to 42% for fruit cordial). The preference ratings for coffee likely encompass genetic sensitivity to, and enjoyment of, the stimulation provided by caffeine. However, not only coffee but many other carbonated fizzy beverages contain caffeine, a psychoactive substance that may be a key driver of beverage liking. Gareth and Griffins (1998) suggested the liking for fizzy caffeinated beverages may be reinforced by the invigorating effect of caffeine, and avoidance of withdrawal symptoms, rather than an affinity for the actual flavour of the beverage^25^. These studies suggest that both direct genetic variability in the biological caffeine-response (e.g. polymorphisms in the caffeine metabolism pathway), and the more indirect psychological mechanisms (e.g. sensitivity to caffeine-induced anxiety) might be influencing caffeinated beverage preference scores^26^.

In line with the heritability estimates for SSBs, NNSBs, and fruit cordial observed in the present study, one previous twin study showed variation in liking for ‘sweet and high carbohydrate foods’ which included diet-and non-diet soft beverages and fruit juices, to be moderately heritable (52%) in a large sample of adults (n = 2596)^14^. However, this estimate only provides limited comparability due to sweet foods and beverages being combined in this category.

Sweetness dominates as the underlying taste of SSBs, NNSBs, fruit cordial, orange juice, and to some extent for the natural sweetness of milk. Finding moderate genetic influence on liking for non-alcoholic beverages may in part reflect genetically-determined sensitivity or preference for sweetness. A previous twin study found that sweetness sensitivity for two different sugars (glucose and fructose) and two different non-nutritive sweeteners (aspartame and neohesperidine dihydrochalcone), was largely explained by a common genetic pathway underlying them all; this suggests a genetically-determined predisposition towards liking for sweet flavours in general^27^. In addition, a family study found that the pleasantness ratings for a strong sweet solution (41%), the pleasantness rating (40%) and frequency of consumption (50%) of sweet foods, and craving for sweet foods (31%) were all moderately heritable^28^. To date no genome-wide association study has been undertaken to identify common genetic variants associated with sweetness preference, but this is likely the result of the limited availability of data on sweetness preference measures in large samples with genome-wide genotyping. However, polymorphisms in the sweet taste receptor genes T1R2 and T1R3 have been associated with variation in sugar consumption^29^ and sucrose taste sensitivity^30^. Evidence from a mouse study suggests that sweetness perception is reliant on the functionality of taste receptor subunits T1R1/T1R3. Zhao *et al*. (2003) observed that mice knockout models for these genes fail to respond to stimulation by sweetness^31^, implicating genetic variability in these genes as plausible contributors to the observed genetic basis of beverage preference scores.

The liking scores for tea and coffee in this study enquired about their unsweetened variety, meaning that these were the only non-sweet beverages considered. Evidence from a recent twin study (n = 1901; mean age: 16.2 y) found considerable correlations (r = 0.35–0.40) between the mean perceived intensity of a “sweet factor” (comprising intensity ratings for glucose, fructose, neohesperidine dihydrochalcone, and aspartame) and perception intensity of three bitter solutions (sucrose octa-acetate, quinine, and caffeine). The correlations were largely explained by shared genetic influences, with 8% of sweetness perception and 17–37% of the bitterness perception ratings attributable to genes common to both traits. In addition, the magnitude of the heritability of sweetness perception (36%) and bitterness perception (35–40%) were at a similar level^32^. The results from this study agree with our findings that heritability estimates did not differ significantly between sweet beverage types (18–42% for SSBs and NNSBs) and non-sweet types (29–41% for tea and coffee). However, it is important to note that the sample sizes were slightly lower for coffee and tea as the mean scores for this group only included those participants who reported drinking these hot beverages, and this may have influenced the heritability estimates for the liking of these non-sweet beverages.

Variation in preferences for beverages was influenced strongly by individual environmental factors (e.g. friends, lifestyle choices), and less so by genetic factors (shared environmental factors were undetectable). This was especially the case for orange juice, coffee and milk, for which 82%, 71%, and 68% of variation in preference scores were explained by unique environmental factors, respectively. The strong influence of the unique (versus shared) environment, reflects the lifestyle changes experienced by adolescents. Adolescence is a period of transition towards independence and autonomy, with greater time spent interacting with peers outside the home, and conforming to perceived societal- and peer pressures. In this respect, (social) media and the commercial food and beverage environment may start to replace family rules and habits (learned at home), exerting a stronger influence on food and beverage choices. While young adults are experiencing these changes, it is important to consider that these are embedded in a wider societal context. For instance, indicators of lower SES have previously been identified as determinants of higher SSB intake and intake of dietary energy from sweetened beverages, overall^7,33^. The association between lower SES and higher SSB consumption has been demonstrated in high-income countries such as the UK and USA^7,34^. No equivalent study has yet investigated the role of SES influencing non-alcoholic drink preferences. In our study, we found no effect of the shared environment but this does not mean that SES is unimportant. As has been previously suggested in an elegant piece of work by Plomin (2011), growing up in a shared household may be experienced differently by each individual living in that same household. As such, the unique experience of SES may be contributing to the substantial unique environmental influence on beverage preferences seen in our study, even though SES intuitively may be considered a shared-environmental factor^35^.

During adolescence, individuals likely begin to diversify the food and drinks they consume, while developing an appreciation for more complex flavours (e.g. the bitterness in alcoholic beverages, vegetables)^36^. Interestingly, variation in beverage preferences appeared to have a slightly higher environmental influence than variation in food preferences, measured in this sample at the same age^11^. For instance, the present study demonstrated a relatively low estimate for the heritability of orange juice preference (18%) compared to fruit preference (49%) (which included the liking for oranges). This suggests that liking of beverages does not simply reflect flavour but also includes factors such as texture, intensity of sweetness, effort involved in consumption etc.^37^.

Perhaps more importantly, our results indicate that beverage preferences may have even greater potential for environmental modification than food preferences. This is encouraging given the concern regarding high intake of SSBs, and may reflect the potent marketing strategies of SSB manufacturers in the UK. However, other macro-level factors such as SES and the precise mechanism by which these determinants affect SSB intake and preferences will require more research if environmental intervention is to shift drink choices towards healthier options.

### Strengths and limitations

Although this is the first study to examine the aetiology of a range of non-alcoholic beverage preferences, only a limited number of beverage types were included in the questionnaire. In particular, sports beverages, energy drinks, and smoothies were not included, although adolescents are by far the most frequent consumers, and the largest growing consumer group of energy and sports drinks^3,38^. In addition, the measure of liking of fruit cordial did not distinguish between sugar- or artificially-sweetened fruit cordial. However, as this was the first study to investigate preference for non-alcoholic beverage types, these broad beverage preference categories will serve as a base to inform future studies which aim to investigate beverage consumption behaviours in more detail. For instance, it would be valuable to investigate the aetiology for the liking of water, which is the beverage type consumed most frequently in this age group^39^.

Preference scores were based on self-report, which is subject to bias; it is possible that adolescents underreported liking for beverages typically considered as “unhealthy”. SSB consumption has previously been found to be underreported by up to 30–40% when validated against an objective blood biomarker^40^, and this type of underreporting has been shown to increase with BMI^41^. However, most participants in this study had a healthy BMI, and the questionnaire data were collected anonymously.

It is possible that beverage preferences are susceptible to short-term variation, influenced by recent consumption or exposure to certain tastes or diets in the period preceding measurement^42,43^. However, the test-rest pilot study of this questionnaire indicated that beverage preference scores were reasonably reliable over a two-week period.

We only identified two twin studies that had investigated genetic and environmental influence on beverage preferences, and both examined coffee only. Further research is therefore needed to replicate our findings, and to establish if the aetiology of beverage preferences varies with developmental stage (e.g. toddlerhood, early childhood or adulthood). This is especially warranted given that energy consumed from beverages now accounts for a substantial proportion of energy intake in the modern diet, with roughly 20% of people’s daily calories coming from beverages^44,45^. The sample were predominantly white British and lean, so the results need replicating in more diverse samples. Likewise, we were not able to assess whether the drink preferences of this sample were comparable to the preferences of the UK general population. Rates of underweight (10%) were higher in this subsample of TEDS, and rates of overweight (13.4%) and obesity (4.5%) were lower, relative to the UK population for this age group (underweight = 8%; overweight = 21%; obese = 16%)^46^. This may perhaps indicate that they may not entirely be representative in terms of dietary factors because they were considerably leaner. Nevertheless, participants were drawn from the TEDS study which has been shown to be reasonably representative in terms of important sociodemographic characteristics of the UK general population^47^. While these indicators do not include the primary variables under consideration, this observation suggests these findings were obtained from a sample representative of the UK’s young adult (18–19 y.) population. Nevertheless, caution is warranted before the results of the study are generalised to larger, more diverse populations.

However, the large sample size and narrow age-range were strengths that allowed reliable estimates for beverage preferences to be established for an important developmental phase.

It was unknown whether the twins had already left home or were residing in a shared home at the time of data collection. The twin method relies on the assumption that MZs and DZs share their environments to a very similar extent, so if more MZs lived together than DZs (or had more frequent contact), the MZ similarity could be inflated, artificially inflating heritability. Although we were unable to rule this out, previous studies have tested the equal environments assumption and found it to be valid^48^.

Lastly, focusing on beverage *preferences* rather than beverage *intake* or *consumption frequency*, has several advantages. Arguably, beverage *preferences* can be measured more accurately than actual intake, using a preference questionnaire that includes a comprehensive list of beverage types distributed to a large population sample; overcoming the high cost and inaccuracies of dietary *intake* assessments^49^. Preferences are one of the most important drivers of actual intake and have been identified as useful predictors of nutrition-related disease risk^50^. Understanding the origins of *preferences* therefore provides useful insights into the factors that need to be targeted by public health programmes to modify consumption (e.g. decreasing intake of SSBs).

### Implications and future research

We established that the largest proportion of variation in beverage preferences in late adolescence is determined by factors in the individual environment. This highlights the potential for modification via public health initiatives aimed at the wider environment. At the same time, this suggests that targeting the family home environment is unlikely to modify beverage preferences among adolescents. One might look towards regulating the marketing strategies used to maximise consumption of energy-dense beverages for insights into how to reduce preferences for them. Current national and international obesity policies adopt a child-centric approach, focusing on regulating and restricting the obesogenic influences such as targeted advertisement of energy-dense food and drinks at children^51^. Preventing excessive weight gain in childhood is a public health priority as a high BMI in childhood is a predictor of overweight or obesity in adulthood^52,53^. However, adolescents’ rates of obesity are rising more rapidly compared to the rest of the adult population^54^, and maintenance of energy balance and healthier dietary intake in this age group also has great potential for disease prevention^55^. While children are rightly considered vulnerable members of society^56^, it is short sighted to assume that once a child transitions into ‘adolescence’ they should be fully exposed to the unregulated commercial pressures of the permissive food and drink environment. Arguably, adolescence is an especially vulnerable developmental period during which individuals are particularly susceptible to social pressures^57,58^. The food and drink industry employ sophisticated strategies which capitalize on this vulnerability to influence adolescents’ attitudes and consumption intentions of their products^59^. Our finding suggests that there might be further population health to be gained if commercial strategies that target young adults are submitted to the same level of regulation as marketing strategies that target younger children^60^.

Previous interventions aimed at changing preferences for foods and beverages, rather than intake, have almost exclusively been undertaken in children perhaps because children’s behaviours are considered to be more malleable^61,62^. There is also wider support for interventions targeted at children, while interventions aimed at adults can be perceived as the enforcement of ‘nanny state’ policies that restrict personal choice. To date, there are two studies providing experimental evidence of adult sweetness preference modification^63,64^. Both studies suggest that self-imposed reduction of sugar and sweetener intake over the course of 2 weeks^63^ to 3 months^64^ has the potential to reduce preferred sweetness levels in food and beverages. Future experimental studies are needed to test the feasibility of such an approach specifically aimed at the modification of sweetness intensity preferences in beverages, and if this taste preference modification can be maintained in the long-term.

## Conclusion

Beverage preferences in this sample were found to be under low to moderate genetic influence, and were not influenced by aspects of the environment completely shared by adolescent twin pairs. This suggests that children’s early shared family experiences may not have lasting effects on beverage preferences but further research will be required to establish whether shared environmental influences may become important again in later life. Reducing the consumption of free sugar at the population-level has in the past years moved to the top of the public health agenda, culminating in the revision of the recommended total daily energy intake from free sugars to <10% by the WHO^65^. At the same time, decreasing obesity levels remain a priority for governments worldwide. Beverages, especially SSBs and other energy-dense beverages are a major contributor to both problems, and yet population consumption levels of these types remain high. Our study emphasizes the importance of unique environmental factors in shaping non-alcoholic beverage preferences, highlighting the potential to modify preferences for unhealthy beverages by national public health programs. Specifically, for SSBs, which have the strongest evidence base for their detrimental health effects, and which can be relatively easily targeted by taxation strategies or levies, policies targeting the wider environment are promising techniques to achieve substantial population level health gains^66^. With the recent rise of national governments and jurisdictions worldwide enacting these fiscal measures, the results from the present study suggest these initiatives might be particularly effective in adolescent populations.

## Electronic supplementary material

Supplemental materials
