# Supplementary material for: The individual environment, not the family is the most important influence on preferences for common non-alcoholic beverages in adolescence
Source: Sci Rep. 2017 Dec 4;7:16822. doi: 10.1038/s41598-017-17020-x (PMC5715117; doi:10.1038/s41598-017-17020-x)
Supplement: Supplementary file 1 — Supplemental materials [file 41598_2017_17020_MOESM1_ESM.pdf]

Online-only supplemental materials

The individual environment, not the family is the most important influence on preferences for common non-alcoholic beverages in adolescence

Andrea D Smith, Alison Fildes, Suzanna Forwood, Lucy Cooke, and Clare Llewellyn

Supplemental Table 1 Model fit and parameter estimates for the saturated, ADE model and submodels of beverage preferences

Beverage type	Additive genetic effect (A)	Dominant genetic effect (D)	Nonshared environment effect (E)	-2LL[3]	Df[3]	AIC[3]	Δ -2LL	p-value
**SSBs[3]**								
Sat				9609.825	2832	3945.825		
**ADE[1]**	**0.30 (0.24, 0.43)**	**0.08 (0.00, 0.17)**	**0.62 (0.55, 0.70)**	**9614.131**	**2835**	**3944.131**	**4.305359**	**0.23**
AE[2]	0.39 (0.32, 0.46)	-	0.61 (0.54, 0.68)	9616.851	2836	3944.851	2.719993	0.10
DE[2]	-	0.24 (0.19, 0.29)	0.76 (0.71, 0.81)	9631.547	2836	3959.547	17.416468	<0.001
E[2]	-	-	1.00 (1.00, 1.00)	9703.572	2837	4029.572	89.441158	<0.001
**NNSBs[3]**								
Sat				9545.841	2818	3909.841		
**ADE[1]**	**0.23 (0.10, 0.36)**	**0.16 (0.07, 0.25)**	**0.61 (0.53, 0.68)**	**9546.322**	**2821**	**3904.322**	**4.804889**	**0.92**
AE[2]	0.43 (0.36, 0.49)	-	0.57 (0.51, 0.64)	9558.221	2822	3914.221	11.8991220	<0.001
DE[2]	-	0.28 (0.23, 0.33)	0.72 (0.67, 0.77)	9557.576	2822	3913.576	11.2537105	<0.001
E[2]	-	-	1.00 (1.00, 1.00)	9660.699	2823	4014.699	114.3772631	<0.001
**Orange juice**								
Sat				7844.431	2840	2164.431		
ADE[1]	0.24 (0.18, 0.32)	0.00 (0.00, 0.04)	0.76 (0.68, 0.83)	7854.672	2843	2168.672	10.241	0.02
**AE[2]**	**0.24 (0.20, 0.32)**	**-**	**0.76 (0.68, 0.80)**	**7854.672**	**2844**	**2166.672**	**0**	**1.00**
DE[2]	-	0.08 (0.03, 0.14)	0.92 (0.86, 0.97)	7873.266	2844	2185.266	18.594	<0.001
E[2]	-	-	1.00 (1.00, 1.00)	7881.523	2845	2191.523	26.851	<0.001
**Fruit cordial**								
Sat				8031.215	2838	2355.215		
**ADE[1]**	**0.28 (0.15, 0.41)**	**0.14 (0.05, 0.23)**	**0.58 (0.51, 0.65)**	**8034.727**	**2841**	**2352.727**	**3.511726**	**0.32**
AE[2]	0.44 (0.38, 0.51)	-	0.56 (0.49, 0.62)	8043.012	2842	2359.012	8.285402	<0.001
DE[2]	-	0.29 (0.24, 0.34)	0.71 (0.66, 0.76)	8051.672	2842	2367.672	16.944929	<0.001
E[2]	-	-	1.00 (1.00, 1.00)	8157.514	2843	2471.514	122.786607	<0.001
**Milk**								
Sat				7269.105	2698	1873.105		
ADE[1]	0.36 (0.21, 0.44)	0.00 (0.00, 0.11)	0.64 (0.56, 0.72)	7277.283	2701	1875.283	8.17838241	0.04
**AE[2]**	**0.36 (0.28, 0.44)**	**-**	**0.64 (0.56, 0.72**	**7277.284**	**2702**	**1873.284**	**0.00117151**	**0.97**
DE[2]	-	0.20 (0.14, 0.26)	0.80 (0.74, 0.86)	7298.526	2702	1894.526	21.24340108	<0.001
E	-	-	1.00 (1.00, 1.00)	7340.874	2703	1934.874	63.59133945	<0.001
**Tea**								
Sat				7109.816	2409	2291.816	11.63	
**ADE[1]**	**0.51 (0.42, 0.58)**	**0.00 (0.00, 0.03)**	**0.49 (0.42, 0.58)**	**7109.816**	**2410**	**2289.816**	**0**	**1.00**
AE[2]	0.50 (0.42, 0.58)	-	0.50 (0.42, 0.58)	7171.829	2410	2351.829	62.01	<0.001
DE[2]	-	0.19 (0.12, 0.26)	0.81 (0.74, 0.88)	7201.412	2411	2379.412	91.6	<0.001
E	-	-	1.00 (1.00, 1.00)	7201.412	2411	2379.412	91.6	<0.001
**Coffee**								
Sat				6351.94	1896	2559.94		
ADE[1]	0.33 (0.14, 0.44)	0.00 (0.00, 0.12)	0.67 (0.56, 0.79)	6356.35	1899	2558.35	4.41	0.22
**AE[2]**	**0.33 (0.21, 0.44)**	**-**	**0.67 (0.56, 0.79)**	**6356.35**	**1900**	**2556.35**	**0.00**	**1.00**
DE[2]	-	0.17 (0.09, 0.25)	0.83 (0.75, 0.91)	6366.625	1900	2566.625	10.27	<0.001
E	-	-	1.00 (1.00, 1.00)	6382.128	1901	2580.128	25.78	<0.001

Maximum Likelihood Structural Equation Modelling (MLSEM) was used to derive estimates of A, D and E, as well as provide two goodness-of-fit statistics; -2LL and the AIC respectively. The selection of the most parsimonious model was indicated by the lowest absolute value of the AIC and smallest $\Delta\chi 2$.

[1] The full ADE model was nested within the saturated model

[2] Sub-models were nested within the full ADE model

[3] *Abbreviations; - 2LL: -2 times log-likelihood of data, AIC: Akaike Information Criterion (AIC), df: degrees of freedom, NNSBs: Non-nutritive sweetened beverages, SSB: Sugar-sweetened beverages*

Supplemental Table 2 Beverage preference scores and tetrachoric correlations (TTC) by zygosity

Beverage item	n[1] (%)[2]	Mean preference score[3] (SD)	Median preference score[3] (SD)	MZ[4] TCC[4] (95% CI)		DZ[4] TCC[4] (95% CI)	
**SSBs**[4]	2841 (99.2)	3.73 (1.37)	4.00 (1.38)	0.47	(0.34, 0.59)	0.11	(0.00, 0.23)
**NNSBs**[4]	2827 (98.7)	3.64 (1.34)	4.00 (1.33)	0.48	(0.34, 0.59)	0.22	(0.11, 0.34)
**Orange juice**	2849 (99.4)	4.43 (0.97)	5.00 (0.97)	0.23	(0.05 0.39)	0.00	(0.00, 0.09)
**Fruit cordial**	2847 (99.3)	4.23 (1.02)	5.00 (1.02)	0.54	(0.42, 0.64)	0.20	(0.09, 0.31)
**Milk**	2707 (94.5)	4.22 (0.95)	5.00 (0.96)	0.60	(0.48, 0.70)	0.09	(0.02, 0.21)
**Tea**	2415 (84.3)	4.31 (1.08)	5.00 (1.08)	0.73	(0.62, 0.82)	0.04	(-0.09, 0.18)
**Coffee**	1905 (66.5)	3.85 (1.29)	4.00 (1.30)	0.48	(0.30, 0.60)	0.08	(-0.06, 0.25)

[1] Number of observations included in mean and median beverage liking score (excl. observations from individuals that never consume the specific beverage)
[2] Percentage of the full sample that reported occasional consumption of the beverage
[3] Preference scores were rated on a 5-point Likert scale, with a higher score indicating a higher preference for the beverage item.
[4] Abbreviations: TCCs: Tetrachoric Correlations; MZ: Monozygotic; DZ: Dizygotic; NNSBs: Non-nutritive sweetened beverages, SSB: Sugar-sweetened beverages

Supplemental Table 3 Threshold Model fit and parameter estimates for the saturated, ACE model and submodels of beverage preferences

Beverage type	Additive genetic effect (A)	Shared environment effect (C)	Nonshared environment effect (E)	-2LL[3]	Df[3]	AIC[3]	Δ -2LL	p-value
**SSBs[1,3]**								
Sat				3724.058	2830	-1935.94		
ACE[3]	0.42 (0.25, 0.52)	0.00 (0.00, 0.11)	0.58 (0.48, 1.00)	3737.736	2837	-1936.26	13.68	0.06
**AE[4]**	**0.42 (0.30, 0.52)**	**-**	**0.58 (0.48, 1.00)**	**3737.736**	**2838**	**-1938.26**	**0.00**	**1.00**
CE[4]	-	0.26 (0.17, 0.34)	0.74 (0.65, 1.00)	3750.700	2838	-1925.3	12.96	<0.001
E[4]	-	-	1.00 (1.00, 1.00)	3782.678	2839	-1895.32	44.94	<0.001
**NNSBs[1,3]**								
Sat				3510.043	2816	-2121.957		
ACE[3]	0.47 (0.15, 0.58)	0.00 (0.00, 0.24)	0.53 (0.42, 1.00)	3515.951	2823	-2130.05	5.91	0.55
**AE[4]**	**0.47 (0.41, 0.58)**	**-**	**0.53 (0.42, 1.00)**	3515.951	2824	-2132.05	0.00	1.00
CE[4]	-	0.33 (0.24, 0.41)	0.67 (0.59, 1.00)	3523.844	2824	-2124.16	7.89	<0.001
E[4]	-	-	1.00 (1.00, 1.00)	3572.634	2825	-2077.37	56.68	<0.001
**Orange juice[2]**								
Sat				2751.467	2495	-2238.53		
ACE[3]	0.23 (0.05, 0.39)	0.00 (0.00, 0.09)	0.77 (0.61, 0.95)	2902.347	2502	-2101.65	150.88	0.00
**AE[4]**	**0.23 (0.05, 0.39)**	**-**	**0.77 (0.61, 0.95)**	**2902.347**	**2503**	**-2103.65**	**0.00**	**1.00**
CE[4]	-	0.07 (0.00, 0.19)	0.92 (0.81, 1.00)	2907.614	2503	-2098.39	5.27	0.02
E[4]	-	-	1.00 (1.00, 1.00)	2908.933	2504	-2099.07	6.59	0.04
**Fruit cordial[2]**								
Sat				3868.794	2836	-1803.21		
ACE[3]	0.51 (0.40, 0.61)	0.00 (0.00, 0.14)	0.49 (0.39, 0.60)	3874.622	2843	-1811.38	5.83	0.56
**AE[4]**	**0.51 (0.40, 0.61)**	**-**	**0.49 (0.39, 0.60)**	**3874.622**	**2844**	**-1813.38**	**0.00**	**1.00**
CE[4]	-	0.33 (0.25, 0.41)	0.67 (0.59, 1.00)	3889.705	2844	-1798.30	15.08	<0.001
E[4]	-	-	1.00 (1.00, 1.00)	3946.426	2845	-1743.57	71.80	<0.001
**Milk[2]**								
Sat				3671.885	2696	-1720.12		
ACE[3]	0.51 (0.39, 0.61)	0.00 (0.00, 0.07)	0.49 (0.39, 1.00)	3685.956	2703	-1720.04	14.07	0.05
**AE[4]**	**0.51 (0.39, 0.61)**	**-**	**0.49 (0.39, 1.00)**	**3685.956**	**2704**	**-1722.04**	**0.00**	**1.00**
CE[4]	-	0.29 (0.20, 0.38)	0.71 (0.62, 1.00)	3709.498	2704	-1698.50	23.54	<0.001
E[4]	-	-	1.00 (1.00, 1.00)	3749.390	2705	-1660.61	63.43	<0.001
**Tea[2]**								
Sat				3142.318	2404	-1665.68		
ACE[3]	0.62 (0.50, 1.00)	0.00 (0.00, 0.06)	0.38 (0.27, 1.00)	3164.124	2411	-1657.88	21.81	0.00
**AE[4]**	**0.62 (0.50, 1.00)**	**-**	**0.38 (0.27, 1.00)**	**3164.124**	**2412**	**-1659.88**	**0.00**	**1.00**
CE[4]	-	0.34 (0.24, 0.43)	0.66 (0.57, 1.00)	3198.295	2412	-1625.71	34.17	<0.001
E[4]	-	-	1.00 (1.00, 1.00)	3240.230	2413	-1585.77	76.11	<0.001
**Coffee[1]**								
Sat				2541.410	1894	-1246.59		
ACE[3]	0.41 (0.25, 0.56)	0.00 (0.00, 0.01)	0.59 (0.00, 1.00)	2548.027	1901	-1253.97	6.62	0.47
**AE[4]**	**0.41 (0.25, 0.56)**	**-**	**0.59 (0.00, 1.00)**	**2548.027**	**1902**	**-1255.97**	**0.00**	**1.00**
CE[4]	-	0.25 (0.12, 0.36)	0.75 (0.64, 1.00)	2555.393	1902	-1248.61	7.37	0.01
E[4]	-	-	1.00 (1.00, 1.00)	2570.613	1903	-1235.39	22.59	<0.001

Maximum Likelihood Structural Equation Modelling (MLSEM) was used to derive estimates of A, C and E, as well as provide two goodness-of-fit statistics; -2LL and the AIC respectively. The selection of the most parsimonious model was indicated by the p=value and the lowest absolute value of the AIC.

[1] Preference score were split by the median value as =<4 vs >4

[2] Preference score were split by the median value as =<5 vs >=5

[3] The full ACE model was nested within the saturated model

[4] Sub-models were nested within the full ACE model

[5] *Abbreviations; - 2LL: -2 times log-likelihood of data, df: degrees of freedom, AIC: Akaike Information Criterion (AIC), NNSBs: Non-nutritive sweetened beverages, SSB: Sugar-sweetened beverages*

Supplemental Table 4 Drink preference score intraclass correlations (ICC) by zygosity and sex

Drink item (n[1])	MZ[2] ICC[2] (95% CI) MM		MZ[2] ICC[2] (95% CI) FF		DZ ICC[2] (95% CI) MM		DZ[2] ICC[2] (95% CI) FF		DZ[2] ICC[2] (95% CI) os	
**SSBs** (n=2841)	0.294	(0.145, 0.429)	0.414	(0.314, 0.504)	0.265	(0.102, 0.412)	0.207	(0.093, 0.314)	0.000	(-0.106, 0.104)
**NNSBs** (n=2827)	0.363	(0.220, 0.489)	0.408	(0.307, 0.500)	0.214	(0.045, 0.368)	0.306	(0.198, 0.403)	0.162	(0.061, 0.258)
**Fruit squash** (n=2847)	0.359	(0.202, 0.494)	0.440	(0.346, 0.526)	0.234	(0.057, 0.393)	0.162	(0.057, 0.263)	0.271	(0.160, 0.371)
**Orange juice** (n=2849)	0.111	(-0.042, 0.258)	0.316	(0.207, 0.415)	-0.020	(-0.190, 0.152)	0.005	(-0.105, 0.114)	-0.041	(-0.146, 0.066)
**Milk** (n=2707)	0.263	(0.113, 0.400)	0.431	(0.322, 0.527)	-0.043	(-0.230, 0.147)	0.126	(0.012, 0.235)	0.040	(-0.090, 0.168)
**Tea**	0.111	(-0.042, 0.258)	0.316	(0.207, 0.415)	-0.020	(-0.190, 0.152)	0.005	(-0.105, 0.114)	-0.041	(-0.146, 0.066)
**Coffee**	0.263	(0.113, 0.400)	0.431	(0.322, 0.527)	-0.043	(-0.230, 0.147)	0.126	(0.012, 0.235)	0.040	(-0.090, 0.168)

Preference scores were rated on a 5-point Likert scale, with a higher score indicating a higher preference for the drink item.

[1] Number of observations included in mean drink liking score (excl. observations from individuals that never consuming the specific drink)

[2] Abbreviations: ICCs: Intraclass Correlations; MZ: Monozygotic; DZ: Dizygotic; FF: same sex female pairs only; MM: same sex male pairs only; os: opposite-sex pairs only

Supplemental Table 5 Parameters estimates (95% Confidence intervals) for A, C and E for males and females considering qualitative and quantitative sex differences for the liking for sugar-sweetened beverages

**Model**	**Male**			**Female**				
	**A_m[1]**	**C_m[1]**	**E_m[1]**	**A_f[1]**	**C_f[1]**	**E_f[1]**	**r_A[1]**	**r_C[1]**
**Full sex limitation (r_A=free)**	0.11 (0.00-0.48)	0.26 (0.04-0.44)	0.63 (0.51-0.76)	0.36 (0.12-0.48)	0.04 (0.00-0.24)	0.60 (0.50-0.68)	0.24 (0.00-0.50)	1.00
**Full sex limitation (r_C=free)**	0.09 (0.00-0.37)	0.28 (0.06-0.44)	0.63 (0.51-0.76)	0.35 (0.08-0.48)	0.05 (0.00-0.27)	0.60 (0.52-0.69)	0.5	1.00 (0.33-1.00)
**Common effects model (rA=0.5, rc=1)**	0.09 (0.00-0.37)	0.28 (0.06-0.45)	0.63 (0.51-0.76)	0.35 (0.12-0.48)	0.05 (0.00-0.24)	0.60 (0.52-0.69)	0.5	1.00
	**A**		**C**		**E**		**Scalar**	
**Scalar Model**	0.35 (0.27-0.42)		0.00 (0.00-0.06)		0.65 (0.58-0.73)		1.09 (1.03-1.15)	
	**A**		**C**		**E**		**r_A**	**r_C**
**Homogeneity model (no sex differences)**	0.35 (0.27-0.42)		0.00 (0.00-0.06)		0.65 (0.58-0.73)		0.5	1.00

[1] Abbreviations: A: additive genetic component of variance; C: shared environmental component of variance; E: unique environmental component of variance; r_A: genetic correlation, r_c : shared environmental correlation, r_E : non-shared environmental correlation.

Supplemental Table 5.1 Heterogeneity model fit statistics for the liking of sugar-sweetened beverages

**Model**		**Comparison**	**Ep**[1]	**-2LL**[1]	**df**[1]	**AIC**[1]	***Δ*-2LL**	**Δdf**	**p-value**	***Δ*AIC**
1	Saturated model		23	9626.144	2818	3990.144				
2	Full sex limitation (rA=free)	1	9	9699.952	2832	4035.952	73.808	14	<0.001	-45.808
3	Full sex limitation (rC=free)	1	9	9699.952	2832	4035.952	73.808	14	<0.001	-45.808
**4**	**Common effects model (rA=0.5, rC=1)**	**2 & 3**	**8**	**9699.952**	**2833**	**4033.952**	**0.000**	**1**	**1.00**	**-2.000**
5	Scalar Model	4	6	9722.293	2835	4052.293	22.341	2	<0.001	-18.341
6	Homogeneity model (no sex differences)	5	5	9732.027	2836	4060.027	9.734	1	0.002	-7.734

Supplemental Table 6 Parameters estimates (95% Confidence intervals) for A, C and E for males and females considering qualitative and quantitative sex differences in the liking for non-nutritive sweetened beverages

Model	Male			Female				
	A_m[1]	C_m[1]	E_m[1]	A_f[1]	C_f[1]	E_f[1]	r_A[1]	r_C[1]
**Full sex limitation (r_A=free)**	0.34 (0.05-0.50)	0.04 (0.00-0.32)	0.61 (0.50-0.26)	0.18 (0.08-0.46)	0.22 (0.08-0.41)	0.60 (0.51-0.70)	0.5 (0.39-0.50)	1.00
**Full sex limitation (r_C=free)**	0.34 (0.12-0.50)	0.04 (0.00-0.36)	0.61 (0.50-0.76)	0.18 (0.04-0.46)	0.22 (0.08-0.41)	0.60 (0.51-0.70)	0.5	1.00 (0.88-1.00)
**Common effects model (rA=0.5, rc=1)**	0.39 (0.24-0.50)	0.00 (0.00-0.34)	0.61 (0.18-0.26)	0.18 (0.07-0.46)	0.22 (0.10-0.41)	0.60 (0.51-0.70)	0.5	1.00
	**A**		**C**		**E**		**Scalar**	
**Scalar Model**	0.36 (0.15-0.47)		0.04 (0.00-0.19)		0.60 (0.17-0.21)		1.03 (0.97-1.09)	
	**A**		**C**		**E**		**r_A**	**r_C**
**Homogeneity model (no sex differences)**	0.35 (0.15-0.47)		0.05 (0.04-0.19)		0.60 (0.53-0.68)		0.5	1.00

[1] Abbreviations: A: additive genetic component of variance; C: shared environmental component of variance; E: unique environmental component of variance; r_A: genetic correlation, r_c : shared environmental correlation, r_E : non-shared environmental correlation.

Supplemental Table 6.1 Heterogeneity model fit statistics for the liking of non-nutritive sweetened beverages

Model	Comparison	Ep[1]	-2LL[1]	df[1]	AIC[1]	*Δ*-2LL	Δdf	p-value	*Δ*AIC
1 Saturated model		23	9537.307	2804	3929.307				
2 Full sex limitation (rA=free)	1	9	9545.274	2818	3909.274	7.967	14	0.891	20.033
3 Full sex limitation (rC=free)	1	9	9545.274	2818	3909.274	7.967	14	0.891	20.033
4 Common effects model (rA=0.5, rC=1)	2 & 3	8	9545.325	2819	3907.325	0.052	1	0.820	-2.00
5 Scalar Model	4	6	9549.760	2821	3907.760	4.4345	2	0.109	0.435
**6 Homogeneity model (no sex differences)**	**5**	**5**	**9551.006**	**2822**	**3907.006**	**1.2462**	**1**	**0.264**	**-0.754**

Supplemental Table 7 Parameters estimates (95% Confidence intervals) for A, C and E for males and females considering qualitative and quantitative sex differences in the liking for fruit cordial

**Model**	**Male**			**Female**				
	**A_m[1]**	**C_m[1]**	**E_m[1]**	**A_f[1]**	**C_f[1]**	**E_f[1]**	**r_A[1]**	**r_C[1]**
**Full sex limitation (r_A=free)**	0.10<br>(0.36-0.36)	0.28<br>(0.15-0.45)	0.62<br>(0.18-0.78)	0.28<br>(0.11-0.41)	0.12<br>(0.03-0.25)	0.60<br>(0.52-0.69)	0.5<br>(0.00-0.50)	1.00
**Full sex limitation (r_C=free)**	0.38<br>(0.23-0.55)	0.05<br>(0.22-0.36)	0.57<br>(0.45-0.71)	0.42<br>(0.51-0.50)	0.00<br>(0.00-0.16)	0.58<br>(0.50-0.67)	0.5	1.00<br>(0.88-1.00)
**Common effects model Both fixes (rA=0.5, rc=1)**	0.38<br>(0.23-0.55)	0.05<br>(0.22-0.36)	0.57<br>(0.45-0.71)	0.42<br>(0.51-0.50)	0.00<br>(0.00-0.15)	0.58<br>(0.50-0.67)	0.5	1.00
	**A**		**C**		**E**		**Scalar**	
**Scalar Model**	0.32<br>(0.49-0.67)		0.00<br>(0.15-9.21)		0.68<br>(0.17-0.21)		-1.17<br>(-1.24-; -1.11)	
	**A**		**C**		**E**		**r_A**	**r_C**
**Homogeneity model (no sex differences)**	0.42<br>(0.36-0.49)		0.00<br>(0.00-0.15)		0.58<br>(0.52-0.64)		0.5	1.00

[1] Abbreviations: A: additive genetic component of variance; C: shared environmental component of variance; E: unique environmental component of variance; r_A: genetic correlation, r_c : shared environmental correlation, r_E : non-shared environmental correlation.

Supplemental Table 7.1 Heterogeneity model fit statistics for the liking of fruit cordial

**Model**	**Comparison**	**Ep**[1]	**-2LL**[1]	**df**[1]	**AIC**[1]	**Δ-2LL**	**Δdf**	**p-value**	**ΔAIC**
1 Saturated model		23	7980.13	2824	2332.13				
2 Full sex limitation (rA=free)	1	9	8001.96	2838	2325.96	21.829	14	0.082	-6.17
3 Full sex limitation (rC=free)	1	9	8001.96	2838	2325.96	21.829	14	0.082	-6.17
**4 Common effects model (rA=0.5, rC=1)**	**2 & 3**	**8**	**8001.96**	**2839**	**2323.96**	**0**	**1**	**1.00**	**-2.00**
5 Scalar Model	4	6	8063.671	2841	2381.67	61.7113	2	<0.001	-57.71
6 Homogeneity model (no sex differences)	5	5	8038.078	2842	2354.08	-25.593	1	1.00	-27.59

Supplemental Table 8 Parameters estimates (95% Confidence intervals) for A, C and E for males and females considering qualitative and quantitative sex differences in the liking for orange juice

Model	Male			Female				
	A_m[1]	C_m[1]	E_m[1]	A_f[1]	C_f[1]	E_f[1]	r_A[1]	r_C[1]
**Full sex limitation (r_A=free)**	0.11 (0.00-0.27)	0.00 (0.00-0.16)	0.89 (0.73-1.00)	0.25 (0.10-0.35)	0.00 (0.00-0.10)	0.75 (0.65-0.85)	0.00 (0.00-0.50)	1.00
**Full sex limitation (r_C=free)**	0.07 (0.00-0.23)	0.01 (0.00-0.16)	0.92 (0.77-1.0)	0.25 (0.13-0.35)	0.00 (0.00-0.09)	0.75 (0.65-0.85)	0.5	1.00 (0.00-1.00)
**Common effects model (rA=0.5, rc=1)**	0.07 (0.00-0.23)	0.01 (0.00-0.16)	0.92 (0.77-1.0)	0.25 (0.13-0.35)	0.00 (0.00-0.09)	0.75 (0.65-0.85)	0.5	1.00
	**A**		**C**		**E**		**Scalar**	
**Scalar Model**	0.17 (0.08-0.25)		0.00 (0.00-0.05)		0.83 (0.75-0.91)		1.11 (1.05-1.17)	
	**A**		**C**		**E**		**r_A**	**r_C**
**Homogeneity model (no sex differences)**	0.18 (0.09-0.25)		0.00 (0.00-0.04)		0.82 (0.74-0.90)		0.5	1.00

[1] Abbreviations: A: additive genetic component of variance; C: shared environmental component of variance; E: unique environmental component of variance; r_A: genetic correlation, r_c : shared environmental correlation, r_E : non-shared environmental correlation.

Supplemental Table 8.1 Heterogeneity model fit statistics for the liking of orange juice

Model	Comparison	Ep[1]	-2LL[1]	df[1]	AIC[1]	Δ-2LL	Δdf	p-value	ΔAIC
1 Saturated model		23	7794.922	2826	2142.922				
2 Full sex limitation (rA=free)	1	9	7843.993	2840	2163.993	49.07021	14	<0.001	21.071
3 Full sex limitation (rC=free)	1	9	7843.993	2840	2163.993	49.07021	14	<0.001	21.071
**4 Common effects model (rA=0.5, rC=1)**	**2 & 3**	**8**	**7843.993**	**2841**	**2161.993**	**0.00**	**1**	**1**	**-2.00**
5 Scalar Model	4	6	7852.797	2843	2166.797	8.804509	2	0.012	4.804
6 Homogeneity model (no sex differences)	5	5	7866.917	2844	2178.917	14.11965	1	<0.001	12.120

Supplemental Table 9 Parameters estimates (95% Confidence intervals) for A, C and E for males and females considering qualitative and quantitative sex differences for the liking for milk

Model	Male			Female				
	A_m[1]	C_m[1]	E_m[1]	A_f[1]	C_f[1]	E_f[1]	r_A[1]	r_C[1]
**Full sex limitation (r_A=free)**	0.26 (0.07-0.41)	0.00 (0.00-0.21)	0.74 (0.59-0.90)	0.38 (0.19-0.47)	0.00 (0.10-0.32)	0.62 (0.14-0.19)	0.5 (0.30-0.50)	1.00
**Full sex limitation (r_C=free)**	0.14 (0.00-0.34)	0.09 (0.00-0.28)	0.78 (0.62-0.94)	0.33 (0.12-0.46)	0.04 (0.00-0.20)	0.63 (0.54-0.73)	0.5	1.00 (0.00-1.00)
**Common effects model (rA=0.5, rc=1)**	0.14 (0.00-0.34)	0.09 (0.00-0.28)	0.78 (0.62-0.94)	0.33 (0.17-0.46)	0.04 (0.00-0.20)	0.63 (0.54-0.72)	0.5	1.00
	**A**		**C**		**E**		**Scalar**	
**Scalar Model**	0.31 (0.22-0.39)		0.00 (0.00-0.05)		0.69 (0.61-0.77)		1.13 (1.07-1.19)	
	**A**		**C**		**E**		**r_A**	**r_C**
**Homogeneity model (no sex differences)**	0.32 (0.22-0.39)		0.00 (0.00-0.05)		0.68 (0.61-0.76)		0.5	1.00

[1] Abbreviations: A: additive genetic component of variance; C: shared environmental component of variance; E: unique environmental component of variance; r_A: genetic correlation, r_c : shared environmental correlation, r_E : non-shared environmental correlation.

Supplemental Table 9.1 Heterogeneity model fit statistics for the liking of milk

Model	Comparison	Ep[1]	-2LL[1]	df[1]	AIC[1]	*Δ*-2LL	Δdf	p-value	*Δ*AIC
1 Saturated model		23	7256.141	2684	1888.141				
2 Full sex limitation (rA=free)	1	9	7319.099	2698	1923.099	62.95796	14	<0.001	45.808
3 Full sex limitation (rC=free)	1	9	7319.099	2698	1923.099	62.95796	14	<0.001	45.808
4 Common effects model (rA=0.5, rC=1)	2 & 3	8	7319.099	2699	1921.099	0.00	1	0.102	2.00
**5 Scalar Model**	**4**	**6**	**7325.083**	**2701**	**1923.083**	**5.984397**	**2**	**0.050**	**1.984**
6 Homogeneity model (no sex differences)	5	5	7344.600	2702	1940.600	19.51656	1	<0.001	17.517

Supplemental Table 10 Parameters estimates (95% Confidence intervals) for A, C and E for males and females considering qualitative and quantitative sex differences for the liking for coffee

Model	Male			Female				
	A_m[1]	C_m[1]	E_m[1]	A_f[1]	C_f[1]	E_f[1]	r_A[1]	r_C[1]
**Full sex limitation (r_A=free)**	0.06 (0.00-0.41)	0.15 (0.04-0.34)	0.79 (0.51-0.95)	0.34 (0.12-0.46)	0.00 (0.00-0.14)	0.66 (0.53-0.81)	0.50 (0.00-0.50)	1.00
**Full sex limitation (r_C=free)**	0.06 (0.00-0.37)	0.15 (0.00-0.34)	0.79 (0.61-0.95)	0.34 (0.12-0.47)	0.00 (0.00-0.15)	0.66 (0.53-0.81)	0.5	1.00 (0.00-1.00)
**Common effects model (rA=0.5, rc=1)**	0.06 (0.00-0.37)	0.15 (0.00-0.34)	0.79 (0.61-0.95)	0.34 (0.12-0.47)	0.00 (0.00-0.14)	0.66 (0.53-0.81)	0.5	1.00
	**A**		**C**		**E**		**Scalar**	
**Scalar Model**	0.29 (0.11-0.39)		0.00 (0.00-0.06)		0.71 (0.61-0.83)		1.06 (1.00-1.14)	
	**A**		**C**		**E**		**r_A**	**r_C**
**Homogeneity model (no sex differences)**	0.29 (0.11-0.39)		0.00 (0.00-0.11)		0.71 (0.61-0.82)		0.5	1.00

[1] Abbreviations: A: additive genetic component of variance; C: shared environmental component of variance; E: unique environmental component of variance; r_A: genetic correlation, r_c : shared environmental correlation, r_E : non-shared environmental correlation.

Supplemental Table 10.1 Heterogeneity model fit statistics for the liking of SSBs

Model	Comparison	Ep[1]	-2LL[1]	df[1]	AIC[1]	Δ-2LL	Δdf	p-value	ΔAIC
1 Saturated model		23	6333.358	1882	2569.358				
2 Full sex limitation (rA=free)	1	9	6349.578	1896	2557.578	16.220	14	0.30	11.78
3 Full sex limitation (rC=free)	1	9	6349.578	1896	2557.578	16.220	14	0.30	11.78
4 Common effects model (rA=0.5, rC=1)	2 & 3	8	6349.578	1897	2555.578	0	1	0.999	2
5 Scalar Model	4	6	6351.370	1899	2553.370	1.7917	2	0.408	2.208
**6 Homogeneity model (no sex differences)**	**5**	**5**	**6355.027**	**1900**	**2555.027**	**3.6571**	**1**	**0.056**	**-1.657**

Supplemental Table 11 Parameters estimates (95% Confidence intervals) for A, C and E for males and females considering qualitative and quantitative sex differences for the liking for tea

Model	Male			Female				
	A_m[1]	C_m[1]	E_m[1]	A_f[1]	C_f[1]	E_f[1]	r_A[1]	r_C[1]
**Full sex limitation (r_A=free)**	0.42 (0.23-0.55)	0.00 (0.00-0.12)	0.58 (0.45-0.74)	0.49 (0.12-0.48)	0.00 (0.00-0.06)	0.51 (0.42-0.62)	0.02 (0.00-0.32)	1.00
**Full sex limitation (r_C=free)**	0.24 (0.00-0.37)	0.10 (0.06-0.44)	0.66 (0.51-0.76)	0.44 (0.28-0.48)	0.03 (0.00-0.27)	0.53 (0.43-0.69)	0.5	1.00 (0.00-1.00)
**Common effects model (rA=0.5, rc=1)**	0.26 (0.00-0.46)	0.09 (0.00-0.29)	0.66 (0.50-0.84)	0.45 (0.28-0.48)	0.02 (0.00-0.13)	0.53 (0.43-0.65)	0.5	1.00
	**A**		**C**		**E**		**Scalar**	
**Scalar Model**	0.41 (0.37-0.50)		0.00 (0.00-0.03)		0.59 (0.50-0.68)		1.05 (0.99-1.11)	
	**A**		**C**		**E**		**r_A**	**r_C**
**Homogeneity model (no sex differences)**	0.41 (0.32-0.50)		0.00 (0.00-0.03)		0.59 (0.50-0.68)		0.5	1.00

[1] Abbreviations: A: additive genetic component of variance; C: shared environmental component of variance; E: unique environmental component of variance; r_A: genetic correlation, r_c : shared environmental correlation, r_E : non-shared environmental correlation.

Supplemental Table 11.1 Heterogeneity model fit statistics for the liking of SSBs

Model	Comparison	Ep[1]	-2LL[1]	df[1]	AIC[1]	Δ-2LL	Δdf	p-value	ΔAIC
1 Saturated model		23	7087.299	2392	2303.299				
2 Full sex limitation (rA=free)	1	9	7130.539	2406	2318.539	43.2395	14	<0.001	-15.24
3 Full sex limitation (rC=free)	1	9	7130.539	2406	2318.539	43.2395	14	<0.001	11.78
4 Common effects model (rA=0.5, rC=1)	2 & 3	8	7129.800	2407	2315.800	0.73889	1	1.00	2.739
5 Scalar Model	4	6	7136.686	2410	2316.686	2.55734	2	0.110	-0.886
**6 Homogeneity model (no sex differences)**	**5**	**5**	**7134.129**	**2409**	**2316.129**	**4.32895**	**1**	**0.115**	**0.557**
